# Boosting the MHC Class II-Restricted Tumor Antigen Presentation to CD4+ T Helper Cells: A Critical Issue for Triggering Protective Immunity and Re-Orienting the Tumor Microenvironment Toward an Anti-Tumor State

**DOI:** 10.3389/fonc.2014.00032

**Published:** 2014-02-18

**Authors:** Roberto S. Accolla, Letizia Lombardo, Rawan Abdallah, Goutham Raval, Greta Forlani, Giovanna Tosi

**Affiliations:** ^1^Department of Surgical and Morphological Sciences, University of Insubria, Varese, Italy

**Keywords:** CIITA, MHC class II, T helper cells, tumor vaccines, anti-tumor immunity

## Abstract

Although the existence of an immune response against tumor cells is well documented, the fact that tumors take off in cancer patients indicates that neoplastic cells can circumvent this response. Over the years many investigators have described strategies to rescue the anti-tumor immune response with the aim of creating specific and long-lasting protection against the disease. When exported to human clinical settings, these strategies have revealed in most cases a very limited, if any, positive outcome. We believe that the failure is mostly due to the inadequate triggering of the CD4+ T helper (TH) cell arm of the adaptive immunity, as TH cells are necessary to trigger all the immune effector mechanisms required to eliminate tumor cells. In this review, we focus on novel strategies that by stimulating MHC class II-restricted activation of TH cells generate a specific and persistent adaptive immunity against the tumor. This point is of critical importance for both preventive and therapeutic anti-tumor vaccination protocols, because adaptive immunity with its capacity to produce specific, long-lasting protection and memory responses is indeed the final goal of vaccination. We will discuss data from our as well as other laboratories which strongly suggest that triggering a specific and persistent anti-tumor CD4+ TH cell response stably modify not only the tumor microenvironment but also tumor-dependent extratumor microenvironments by eliminating and/or reducing the blood-derived tumor infiltrating cells that may have a pro-tumor growth function such as regulatory CD4+/CD25+ T cells and myeloid-derived-suppressor cells. Within this frame, therefore, we believe that the establishment of a pro-tumor environment is not the cause but simply the consequence of the tumor strategy to primarily counteract components of the adaptive cellular immunity, particularly TH lymphocytes.

## Introduction

The onset, expansion, persistence, and spreading of tumors are under the control of a complex series of events that encompass both intrinsic modifications of cancer cells, such as genetic mutations in proto-oncogenes and in tumor suppressor genes, and alteration of the apoptotic process, which cumulatively impact on the homeostasis of the cell cycle, as well as extrinsic mechanisms related to the capacity of the host to counteract tumor growth (1). As far as host mechanisms counteracting the tumor, certainly the immune system plays a major role. The key functions of the immune system consist in the capacity to recognize and fight “foreign aggressors” (the non-self), while sparing self constituents (tolerance), and in conserving specific memory of this event, should foreign aggressors be encountered again (2). Under this view, the genetic modifications affecting tumor cells may lead to the generation of structurally altered or abundantly synthesized proteins that may be seen by the immune system as new antigens or “non-self” products against which the system has not learned to be tolerant during ontogenesis (3). Both innate and adaptive immunity have been shown to participate to this response (4). Nevertheless, the fact that the tumor process takes off in cancer patients demonstrates that the recognition of cancer cells and/or the equilibrium between tumor growth and anti-tumor immune response can be altered, favoring the progression of the malignancy (5, 6). Tumor development and response of the immune system have long been considered opposing actions in the cancer history. However, accumulating data strongly indicate that the two events can synergize, particularly for the tumor prosperity. Tumor cells may, for example, produce mediators that not only counteract the possible protective anti-tumor action of the immune system but also induce immune cells to produce factors or to polarize their functional phenotype in favor of the growth and the expansion of the tumor, thus completely deviating the goal of the immune response (7). Moreover, tumor infiltrating leukocytes, including macrophages (7, 8), regulatory T cells (Tregs) with CD4+/CD25+ phenotype and suppressive function on helper and effector T cells (9), as well as a heterogeneous group of similarly acting myeloid-derived suppressor cells (MDSCs) (10), can cooperate in antagonizing the protective immune response while favoring tumor growth. All together, these observations have created the diffuse belief that a pro-tumor polarization of the innate and adaptive immunity is a sort of inevitable event in the life history of the tumor and a major cause for cancer cells to survive, replicate, and spread.

We have recently discussed a different interpretation of this key issue (11, 12). Indeed, the establishment of a pro-tumor environment may not be the cause but the consequence of the initial inability of the immune system to be efficiently triggered against the tumor. Within this frame, one crucial aspect is the initial recognition of tumor-associated antigens (TAAs) by the adaptive immune system. Two fundamental events must take place in order to initiate the adaptive immune response: (a) antigen processing of complex antigens and presentation of appropriate peptides within the context of MHC class II molecules; (b) recognition of MHC class II-bound peptides displayed on the cell surface by MHC class II-restricted CD4+ T cells, designated T helper (TH) cells (13). TH cells are fundamental for optimal induction of both humoral and cellular effector mechanisms (14), particularly for the maturation of MHC class I-restricted CD8+ naïve T cells, their clonal expansions, and acquisition of cytolytic function (15). The latter function is of relevance in the context of anti-tumor immunity since CD8+ cytolytic T cells (CTLs) are believed to be the major lymphocyte effectors against cancer cells (16).

Initial priming and triggering of naïve antigen-specific TH cells are believed to be mediated by specialized MHC class II-positive cells, particularly dendritic cells (DC), which engulf exogenous antigenic material (pathogens, pathogen debris, cell debris), digest it in appropriate endosomal compartments, degrade the building blocks of life material, that is the proteins, into peptides, and allow the binding of antigenic peptides to MHC class II molecules. The MHC-II–peptide complexes can thus migrate to the cell surface and be displayed for TH cell scrutiny. This general mechanism would imply that endogenously synthesized protein, as tumor antigens are, may not be presented within the context of MHC class II unless they are captured as secreted products or as cell debris-associated components by DC surrounding or infiltrating tumor masses. However, previous elegant studies have shown that endogenous proteins could access the MHC class II pathway of antigen presentation (17, 18) and peptides of these proteins could be recognized and serve as immunogens for TH cell triggering (19, 20).

The majority of tumors, particularly carcinomas, usually do not express MHC class II molecules that are instead expressed by professional antigen presenting cells (APCs) such as DC, macrophages, and B cells (13). Furthermore, although in some circumstances tumor cells may express MHC class II molecules, these cells are believed to act at most as “peptide antigen presenters” to antigen-specific primed TH cells but not as “antigen processors and presenters” for naïve TH cell priming. This is only partially true, since we have demonstrated that, at least *in vitro*, human or murine engineered-tumor cells, stably expressing MHC class II molecules, can process protein antigens and present relevant peptides to MHC-II-restricted antigen-specific T cell clones (21, 22). These results have shown that the intracellular machinery responsible for digesting and exposing MHC-II–peptide complexes on the cell surface for TH cell recognition may work properly also in cells which are not classical APC, and notably in MHC-II-positive tumor cells.

Within this frame, the fact that most tumor cells do not express MHC class II molecules prevents even the possibility that these cells may act as potential APCs for their TAAs and by consequence that they may trigger tumor-specific TH cells.

In this review, we will discuss data from our as well as from other laboratories that strongly suggest how the initial insurgence of the tumor process and the efficient response of the adaptive immune system to the tumor are strongly conditioned by the fact that tumor cells may or may not express MHC class II molecules in the appropriate “physiological” condition, and by the fact that insufficient amount of tumor antigens is offered in a MHC class II-restricted fashion to TH cells.

These two events dramatically influence the formation and the functional role of the tumor microenvironment, conceived not only as tumor tissue but also as tumor-dependent microenvironments at distant sites, particularly in lymphoid tissues of tumor-bearing hosts. We will stress the concept that the generation of a pro-tumor microenvironment is not the cause but the consequence of the failure of the adaptive immune system to recognize MHC-II–tumor peptide complexes by tumor-specific TH cells.

## Induction of Canonical MHC Class II Expression in Tumor Cells may Trigger a Protective Anti-Tumor Immune Response *in vivo*

The idea to promote anti-tumor adaptive immune responses *in vivo* by providing adequate antigen availability (AAA), that means not only sufficient amount of antigen but also access of this antigen to MHC-II binding for optimal triggering of TH cells (12), has been approached from different standpoints. For example, irradiated or genetically modified tumor cells have been used even in clinical trials with the goal to provide host APC with sufficient amount of TAA or to generate, within the tissue injected with tumor cells, a suitable milieu for optimal APC uptake and presentation of tumor antigens by APC via their MHC class II molecules (23). DC loaded with TAAs have been also used with the aim of providing a direct source of ready-to-use MHC-II–tumor peptide complexes for optimal priming and triggering of TH cells (24, 25) and recent clinical results in melanoma patients give further hope in improving clinical responses by this approach (26). Several groups, including ours, have instead investigated the possibility to render tumor cells themselves MHC class II-positive and thus used them as potential surrogate APC for triggering tumor-specific TH cells (27–29). Within this frame, two distinct approaches have been described. The group of Ostrand-Rosenberg induced MHC class II expression in tumor cells by transfecting isolated MHC alpha- and beta chain-encoding genes (28, 30), whereas our group has privileged the transfection of tumor cells with the MHC class II transcriptional activator (CIITA), which is the physiological regulator of expression of all MHC class II genes (31–33). CIITA regulates also the expression of other fundamental genes necessary for MHC-II transport to endosomal compartments and loading of peptides, including the invariant chain (In chain) and DM (34–37). In the first approach, by expressing only isolated MHC class II molecules without expression of In chain, both the district of interaction and the quality of interacting peptides, including tumor-associated peptides, are totally different as compared to the site and the peptides interacting with physiologically expressed MHC class II molecules. The rationale underlying this approach was to allow peptides from TAAs, which are endogenous proteins, to associate with MHC class II molecules in the ER, similarly to what happens for MHC class I-peptide binding, and thus allow better recognition of putative tumor antigens by MHC class II-restricted TH cells. However, although with the SaI sarcoma model (mostly used by the Ostrand-Rosenberg group) protective immunity could be generated *in vivo* by vaccinating mice with MHC-II (alpha-beta)-transfected cells, the cellular correlates of protection remained not completely clarified, because no other tumor models were studied intensively. In the SaI tumor model, it has been suggested that tumor cells may not act directly as surrogate APCs but as donors of peptide–MHC class II complexes for professional APC, such as DC, that in turn stimulate TH cells (30). However, it must be kept in mind that in the absence of In chain hardly any MHC class II molecules are in a stable peptide-loaded form. Cells from In chain knock-out mice show a dramatic reduction in cell surface MHC class II molecules, resulting from both defective association of class II alpha- and beta-chains and markedly decreased post-Golgi transport. The few class II alpha/beta heterodimers reaching the cell surface behave as empty molecules or as molecules occupied by an easily displaced peptide, and display a distinct structure. Moreover spleen cells from these mice are defective in their ability to present intact protein antigens (36, 38).

Our approach based on CIITA-mediated expression of MHC class II molecules as well as upregulation of In chain, seemed to us more prone to mimic the physiological condition by which TAA-derived peptides may be charged onto MHC-II in a more stable way and exposed to the cell surface for TH cell scrutiny. Indeed, it was possible to show that CIITA-transduced tumor cells of at least four distinct histological origin can be efficiently rejected or strongly prevented in their growth when injected into immunocompetent syngeneic mice (22, 27, 39), demonstrating the general applicability of our model system. Furthermore, it was shown that mice vaccinated with CIITA-transfected tumor cells develop an anamnestic response not only against the CIITA-expressing tumor but also, most importantly, against the CIITA-negative parental tumor cells leading to a very efficient rejection of the parental tumor (22, 27, 39). CIITA-dependent MHC class II expression in tumor cells was instrumental to trigger the anamnestic protective immune response against the parental tumor, as also demonstrated by vaccination experiments with non-replicating CIITA-transfected tumor cells (40). The search for cellular correlates of protection highlighted three fundamental points. Firstly, long-lasting immunity was generated in CIITA-vaccinated mice since these animals were able to reject parental tumors after 6 months from initial vaccination. Second, rejection and/or reduced tumor growth were mediated by tumor-specific CD4+ TH and CD8+ CTL, because ablation of these subpopulations *in vivo* by injection of specific anti-CD4 or anti-CD8, but not by anti-B cell or anti-NK cell monoclonal antibodies, prevented the acquisition of protective immunity after injection of CIITA-expressing tumor cells. Third, and of particular relevance, transfer of purified CD4+ T cells from vaccinated mice into naïve recipients was sufficient to confer protection against parental tumors (22, 27, 39, 40).

These results demonstrated that expression of CIITA-mediated MHC class II molecules in tumor cells was instrumental in triggering a protective adaptive immune response in immunocompetent mice. One possible conclusion from these findings was that tumor cells expressing CIITA-dependent MHC-II molecules could serve themselves as APC of their own TAA for initial triggering and priming of tumor-specific TH cells. Alternatively, preformed MHC-II–TAA peptide complexes derived from CIITA-transfected tumor cells could be shed from the cells, captured by professional APC and used by these cells to reach the AAA necessary to trigger an efficient priming of tumor-specific CD4+ TH cells.

## Characterization of the Tumor and Lympoid Organs Microenvironment in CIITA-Tumor Vaccinated Mice and in Parental Tumor-Bearing Mice

To further investigate the crucial issue of the possible direct APC function of CIITA-transfected tumor cells, a series of experiments were undertaken in our laboratory focusing first on the histological characterization of the tumor microenvironment in CIITA-tumor and in parental tumor-injected mice (22). While tumors derived by injecting parental, MHC-II-negative cells showed very poor, if any, infiltration of lymphocytes and scarce presence of macrophage and neutrophils in several tumor models, CIITA-transfected MHC-II positive tumors were rapidly and firstly infiltrated by CD4+ T cells, followed by CD8+ T cells, and later by DC, macrophage, and neutrophils. Already in the early phase of CD4+ and CD8+ T cell infiltration, extensive areas of tumor cell necrosis surrounded by the above T cells and progressively infiltrated by large numbers of neutrophils could be observed. Indeed, the histological picture observed in the tumor microenvironment generated by CIITA-transfected tumor cells was the classical picture of an inflammatory lesion. Most importantly, parental tumor rechallenge of CIITA-vaccinated mice showed a histological aspect of the tumor tissue virtually superimposable to the one observed in naïve mice injected with CIITA-tumor cells. Indeed, in CIITA-tumor vaccinated and parental tumor challenged mice, the lymphocyte infiltration and the tumor necrotic areas were even more massive than in naïve mice injected with CIITA-tumor cells (39).

This finding, along with the capacity of CIITA-expressing tumor cells to process and present antigenic peptides to CD4+ T cells *in vitro* (21, 22), supports the hypothesis that much of the tumor-specific TH cell triggering takes place in the tumor tissue as consequence of the recognition of MHC-II–TAA peptide complexes expressed by CIITA-transfected tumor cells.

If confirmed, this will provide further evidence that tumor cells can act as APC for MHC-II-restricted TH cells in the absence of DC. This in turn will show that alternative mechanisms of antigen presentation for TH cell priming *in vivo* can not only occur but they can occur in milieus other than lymphoid tissues. A further, and in our opinion, extremely important conclusion of these findings is that MHC-II molecules of CIITA-transfected tumor cells are indeed loaded with the relevant immunogenic tumor-derived peptides; these peptides might be eluted out and sequenced to assess the tumor-associated peptidome, a fundamental step for constructing more promising anti-tumor vaccines aimed at optimally stimulating TH cells. Extending the knowledge of the repertoire of MHC-bound tumor-derived peptides by purifying and characterizing the tumor-associated peptidome and choosing the most appropriate cocktail of peptides to prepare anti-tumor vaccines has been shown recently to be a very promising approach for the construction of suitable multi-peptide vaccine in human renal cancer (41).

The increased immunogenicity of the tumor generated by the CIITA-mediated MHC-II expression and the consequent adequate tumor antigen availability at the right time and at the right place affects dramatically the tumor microenvironment, moving the balance from a pro-tumor to an anti-tumor microenvironment. The subversion of the tumor microenvironment affected also the number of Tregs observed in tumor draining lymph nodes. Tregs are believed to play an important role in down-modulating the function of CD4+ TH cells in adaptive immunity, and their number and function were found to increase in tumor-bearing hosts (9). In human colorectal cancer, very recently, the presence of Tregs and the consequent reduced response of anti-tumor CD4+ TH cells has been associated also to the progression of the diseases (42). Accordingly, we found that parental tumor-bearing mice had a substantial increase in the number of Tregs in tumor draining lymph nodes (40), although the relative suppressive capacity of these cells on TH function *in vitro* and on tumor growth *in vivo* was not increased (43). On the other hand, the number of Tregs in CIITA-tumor injected and protected mice was found to be much lower and virtually superimposable to that of naïve mice (40). Thus, initial optimal triggering of the adaptive immune response against the tumor prevented the increase of a crucial cellular component with suppressive function on CD4+ TH cells and with pro-tumor behavior on tumor microenvironment. Active investigation is also focused, at present, on the possibility that CIITA-tumor vaccination may also affect the function and/or the number of other cells, like MDSC, with suppressive function on adaptive anti-tumor immunity, particularly in light of recent findings suggesting that MDSC can act as tolerizing cells toward tumor antigen recognition in specialized sites of the splenic lymphoid tissue (44).

## Anti-Tumor Immunity Elicited after Chemotherapy, Radiotherapy, and Cytokine Therapy: The Concept of Therapy-Induced Anti-Tumor Vaccination

In recent years, it has become apparent that the beneficial effects of various conventional therapeutic modalities, including chemotherapy and radiation therapy, are associated with the rescue of an immune response against the tumor.

Most chemotherapeutic drugs exert their action through the induction of apoptosis, a mechanism that has long been interpreted as generating tolerogenic signals for the immune system. This was generally attributed to the scarce or absent inflammation generated by the apoptotic process and thus by the impossibility to trigger leukocyte migration and lymphocyte activation. However, after the important work of the Novak and colleagues, it appeared that drug-induced apoptotic tumor cells behave as potent “providers” of immunogenic tumor antigens for APC-dependent cross-stimulation of tumor-specific CTL (45). After this observation, many studies have confirmed that not only gemcitabine (the drug initially used by Novak et al.) but also cyclophosphamide, oxaliplatin, cisplatin, etoposide, topotecan, paclitaxel, and vinblastine may increase tumor recognition by the immune system via several mechanism not only related to apoptosis induction but also to the capacity of these drugs to upregulate the expression of tumor antigens and to increase MHC class I expression on tumor cells, thus favoring recognition and triggering of tumor-specific CTL (46, 47). Within this frame, it has been shown that chemotherapy may activate also innate immune effectors such as NK cells (48). The detailed study of the effect of chemotherapy on the anti-tumor immune response has provided new information on the complexity of actions and targets that the treatment brings about. Indeed, chemotherapy may be beneficial not only because it activates immune effectors but also because it counteracts negative regulators of anti-tumor immunity in the tumor microenvironment and at distant sites. In fact, chemotherapy may reduce the infiltration of Tregs (49–51) and may reduce the action and/or decrease the number of MDSC (52, 53).

Similarly to chemotherapy, radiotherapy can be associated to the elicitation of an immune response against the tumor (54). Radiotherapy very often induces an inflammatory reaction. It is widely believed that radiation-mediated inflammation promotes tissue necrosis (54, 55) and necrotic tissue may constitute a major source of tumor antigen for triggering adaptive immune responses. Indeed, radiation can induce antigen release in the tumor microenvironment, consequent activation of APCs and triggering of CTL responses (56, 57). Interestingly, radiotherapy may also influence the activation of tumor-associated DC, disclosing the possibility that potentially tolerized DC may re-acquire strong tumor antigen APC function for immune lymphocyte effectors (58) Moreover, radiation can increase the expression of tumor antigens (59) and the cell surface expression of MHC class I molecules by various modalities. Together, these events increase the potential to present TAA peptides to CTL (60–62). In recent years, it has become apparent that radiation can also induce apoptosis and apoptotic cell death can efficiently support stimulation of adaptive immunity (63, 64) in a similar way to the observed effect of chemotherapy.

Not only classical chemotherapy and radiotherapy may induce triggering of the adaptive immunity, biological therapies such as the ones that tag the tumor tissue with certain anti-tumor cytokines may also be responsible of a strong anti-tumor immunity that in certain cases is indeed the mechanism through which the therapy generates tumor cure and permanent tumor clearance (11, 12, 40, 65). Together with the group of Luciano Zardi and Laura Borsi, we have investigated the role of TNF-alpha and Il-2 cytokines conjugated with an scFv antibody (L19) specific for the extra domain B of fibronectin, selectively expressed in the neovasculature (66). Injection of L19-mouse TNFα conjugate (L19mTNFα) induces a dramatic cell death of established tumors in several murine tumor models including the WEHI-164 fibrosarcoma, C51 colon carcinoma (67), and N2A neuroblastoma (65), because it allows therapeutically active doses of TNFα to be reached at the tumor site in which the cytokine increases intravasal coagulation and trombosis around the tumor, further favoring tumor necrosis. In this approach of targeted tumor therapy, three observations lead us to envisage a crucial participation of the adaptive immune system to the tumor cure: (a) the high rate of complete and long-lasting tumor eradication, (b) the fact that tumor-bearing immunodeficient SCID mice were partially resistant to the L19mTNFα treatment even when combined with the cytostatic drug melphalan, and (c) most importantly, the fact that cured mice were resistant to challenge with parental tumor (67). Indeed, immunological studies demonstrated that cured mice developed both anti-tumor CD8+ and CD4+ T cell immunity. Importantly, adoptive cell transfer of CD4+ T cells from cured mice into naïve recipients was sufficient to protect the animals from tumor take (43, 65, 67). These studies lead us to formulate the concept of *therapy-induced anti-tumor vaccination* because definitive cure of the tumor was achieved after elicitation of a potent adaptive immune response and the treatment was instrumental in reaching protective immunity. The study of the tumor microenvironment in this tumor model was of particular relevance for refining the concept of therapy-induced anti-tumor vaccination. In fact the tumor microenvironment after L19mTNFα alone or in combination with L19mIL-2 showed a marked cellular remodeling with respect to the one observed before therapy. Extensive areas of necrosis infiltrated by granulocytes and macrophages and particularly by CD4+ and CD8+ T cells were observed. The generation of an abundant tumor necrotic material, together with the known effect of TNFα in recruiting DC in inflammatory sites (68) could be a crucial element to generate AAA for fueling professional APC, thus reaching optimal saturation of MHC class II molecule-tumor peptide complexes on DC. These APC could then stimulate specific anti-tumor TH cells and effector CTL, leading to the complete rejection of the tumor and to the establishment of a critical reservoir of memory effector cells responsible for the accelerated rejection of the tumor upon challenge. Again, the therapeutic approach was instrumental in creating the conditions for a protective anti-tumor adaptive immune response responsible of the tumor elimination. The immune-related modification of the tumor microenvironment following therapeutic treatment was also strongly appreciated in the tumor draining lymphonodes and in the spleen. The predominant presence of CD4+ TH2-type cells observed in tumor-bearing hosts before therapy was reconverted in a predominant, although not exclusive, presence of TH1-type cells (43, 65). A further element of remodeling of the tumor microenvironment consisted in the modification of the number, but not the function, of regulatory Tregs with suppressive characteristics on CD4+ TH cells (43). Thus, similarly to the preventive vaccination approach with CIITA-expressing tumor cells, the protective anti-tumor immune response generated by the treatment with L19mTNFα was associated to a rapid appearance and conversion toward a TH1 immune phenotype. This preliminary *in vivo* experimental investigation has paved the way to initiate clinical trials with L19mTNFα and melphalan in patients with selected tumors (69, 70).

## A Hierarchic Model for the Protective Anti-Tumor Immunity

**Figure 1:**
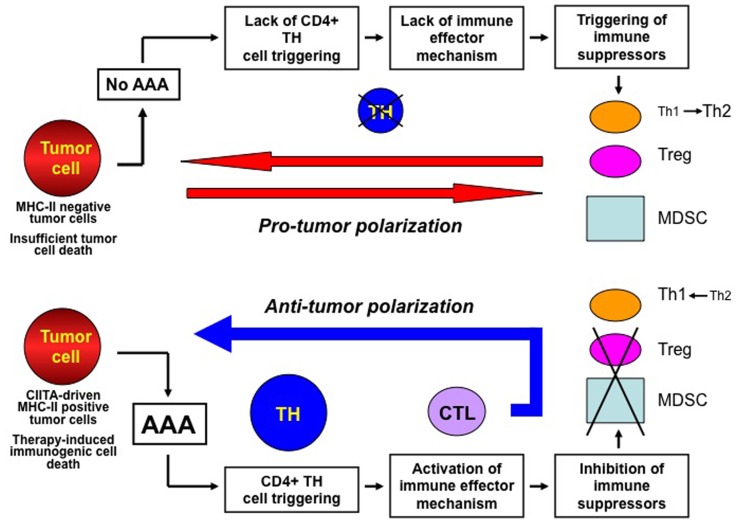
**The key actions for establishing a protective anti-tumor immunity against cancer**. The immune response against cancer is put on break by several mechanisms among which tumor antigen availability and stimulation of MHC class II-restricted CD4+ TH cells are key features (upper part of the Figure). Insufficient tumor antigen availability and/or insufficient MHC-II–tumor peptide complexes (cumulatively defined as adequate antigen availability or AAA) lead to insufficient stimulation of TH cells. This results in lack of immune effector responses which may favor the establishment of immune suppressor mechanism on anti-tumor responses, such as polarization of TH responses toward a TH2 phenotype, activation and increase number of regulatory T cells (Treg) and myeloid-derived-suppressor cells (MDSC), which cumulatively create a pro-tumor polarization of the tumor microenvironment. Presence of AAA (lower part of the Figure) generated, for example, by MHC class II expression in tumor cells or by therapy-induced immunogenic cell death efficiently triggers tumor specific TH cells and this is instrumental to both activate immune effector mechanisms such as CTL and repress and/or prevent suppressor mechanisms on protective immunity. This results in the generation of an anti-tumor microenvironment and in a strong adaptive immune response against the tumor.

**Figure 2:**
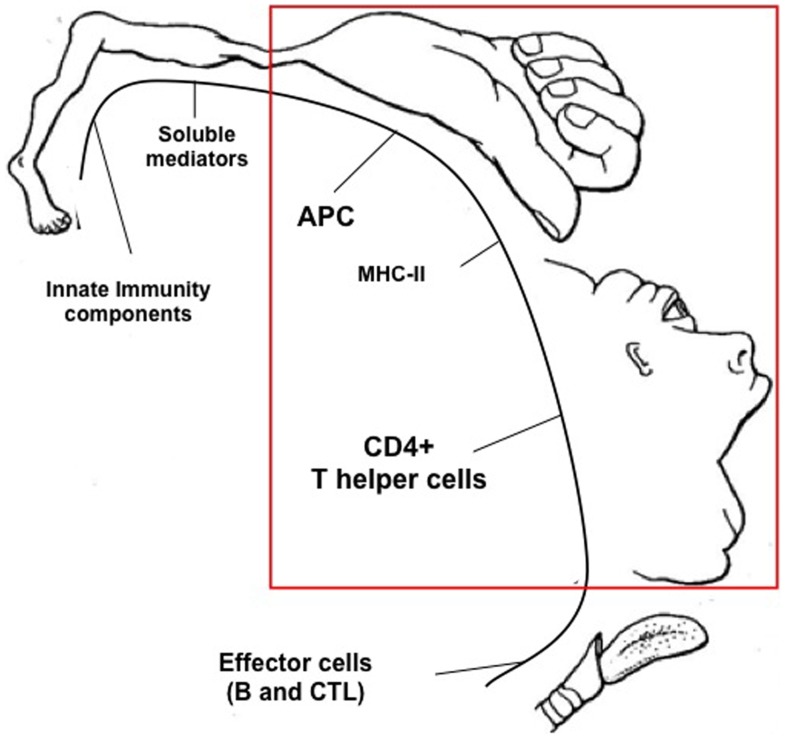
**Proposed hierarchic position of components of the immune system involved in the productive response against cancer**. By similarity with the pictorial representation of the “Cortical Homunculus” of W. Penfield, a hypothetical “Immunological Homunculus” is depicted identifying specific components of the immune system as the hierarchically most important actors against the tumor. These are, *in primis*, the tumor antigen presenting function exerted, for example, by dendritic cells or by tumor cells expressing MHC class II molecules, and the MHC class II-restricted CD4+ T helper cells. Other important components are defined as effector cells, innate immunity components, and soluble mediators.

We have seen how either preventive or therapeutic anti-tumor modalities may generate an anti-tumor immune response that in many cases strongly contribute to the modification of the tumor microenvironment and to the eradication of the neoplastic lesion. These results give strong support to the idea that a correct stimulation of the immune system, particularly of the adaptive arm of it, constitutes a primary strategy to be pursued for combating and defeating cancer (see Figure 1). With the exception of preventive vaccination approaches whose final goal is indeed to primarily trigger the adaptive immune system and generate long-lasting immunity against cancer, other classical therapeutic approaches have unveiled the participation of different components of the immune system to a possible successful outcome upon therapy. Recent excellent reviews have summarized the mechanisms and the distinct components of the immune systems that are involved in such therapeutic approaches (46, 54, 71). However, the many possible players that the immune system may use for either triggering a positive response or counteracting a suppressive response against the tumor are not, in our opinion, equally important for the elicitation of a protective immune response against cancer. Based on our experience and on the results of many groups described in this review, we believe that a crucial and hierarchically predominant step is constituted by the efficacy of MHC class II-restricted tumor antigen presentation to CD4+ TH cells (see Figure 2). We have defined this step as AAA to indicate the optimal tumor antigen dose and related antigen processing and MHC-II-restricted presentation necessary to efficiently trigger tumor-specific TH cells (12). We have shown that AAA can be obtained in several ways either by inducing MHC class II expression in tumor cells by transfection with the AIR-1 gene-encoded MHC CIITA, thus providing functionally sufficient MHC-II–tumor peptide complexes for TH cell scrutiny, or by increasing the availability of tumor antigens by specific treatment of established tumors with L19mTNFα. The fundamental finding of both approaches was the elicitation of long-lived anti-tumor specific TH cells capable to eradicate the tumor and to protect the host against further tumor challenge. The primary importance of AAA in the hierarchic scale of anti-tumor immunity stems also from the fact that the correct triggering of TH cells was sufficient to unleash the chain of events leading to a strong effector CTL response and to the abrogation or, at least, the attenuation of suppressor mechanisms operating on the anti-tumor immune response (Figure 1). Moreover, and importantly, the optimal initiation of the adaptive immune response dictated by AAA was sufficient to reorient the tumor microenvironment from a pro-tumor to an anti-tumor microenvironment. Thus, in a sort of re-edition of the immunological homunculus theory originally proposed by Cohen to explain autoimmunity (72), we propose that most of the protective control exerted by the adaptive immune system on cancer derives from AAA (APC and MHC-II, the hand in Figure 2) and consequent activation of tumor specific TH cells (the face in Figure 2).

By similarity, we believe that classical anti-tumor chemotherapeutic or radiotherapeutic approaches that generate or increase an underlying anti-tumor adaptive immune response are most successful when they can generate AAA and indeed many examples supporting this concept have been described (56, 60, 61, 73).

## Concluding Remarks and Perspective for Vaccination Against Human Tumors

A large number of studies both in animal experimental tumor models and in human tumor-bearing hosts have strongly suggested that anti-tumor immunity can be triggered and/or implemented by several preventive and therapeutical modalities. Can we translate this information to provide in the very near future better anti-tumor treatments and, hopefully, more reliable anti-tumor cure? Spontaneous or experimentally induced HLA class II expression by exogenously transferred CIITA in tumor cells may be instrumental to purify and sequence more immunogenically relevant HLA class II-bound TAA peptides. In conjunction with similarly derived HLA class I-restricted TAA peptides, they will be the basis for the construction of a multi-peptide, multi-epitope vaccine that can target both CD4+ TH and CD8+ CTL anti-tumor responses.

In relation to chemotherapy, radiotherapy, and cytokine-mediated therapy and their effects on anti-tumor immunity, we believe it will be of particular relevance to select new drugs, to apply radiotherapeutic modalities, and to construct cytokine-based compounds, respectively, that may offer the best effects in stimulating AAA and consequent triggering of anti-tumor specific TH cells. This, in turn, will set the ground for the generation of what we have defined as therapy-induced anti-tumor vaccination.

## Conflict of Interest Statement

The authors declare that the research was conducted in the absence of any commercial or financial relationships that could be construed as a potential conflict of interest.
